# Fluorometholone–antibiotic interactions in canine ocular bacteria: *in vitro* susceptibility changes in common corneal infection pathogens

**DOI:** 10.3389/fvets.2026.1860758

**Published:** 2026-06-10

**Authors:** Donghee Kim, Ji Seung Jung, Jiwoo Park, Kyung-Mee Park

**Affiliations:** 1 Laboratory of Veterinary Surgery and Ophthalmology, College of Veterinary Medicine, Chungbuk National University, Cheongju, Republic of Korea; 2 Cheonan Animal Medical Center, Cheonan, Republic of Korea; 3 Ulsan S Animal Medical Center, Ulsan, Republic of Korea; 4 Clear Eye Animal Clinic, Daejeon, Republic of Korea

**Keywords:** antibiotic resistance, bacterial keratitis, corticosteroid interaction, fluorometholone, ophthalmic drug interaction

## Abstract

**Introduction:**

Corticosteroid-antibiotic combinations are commonly used in veterinary ophthalmic practice, yet the potential for corticosteroids to modulate antimicrobial susceptibility remains incompletely characterized. This study evaluated the *in vitro* effects of fluorometholone (FML) on the antimicrobial susceptibility of three major canine ocular pathogens—*Staphylococcus pseudintermedius*, *Streptococcus canis*, and *Pseudomonas aeruginosa*—and compared its interaction profile with that of dexamethasone.

**Methods:**

Thirty clinical isolates from dogs with bacterial keratitis were tested using standardized broth microdilution assays with 17 ophthalmic antibiotics at increasing FML concentrations (0, 0.25, 0.5, and 1 mg/mL). Changes in median minimum inhibitory concentrations (MICs) and susceptibility distributions were analyzed using the Friedman test and Cochran’s *Q* test.

**Results:**

FML exposure produced selective, species- and antibiotic-dependent effects. *S. pseudintermedius* showed significant MIC increases for amikacin, ticarcillin, and tobramycin, with susceptibility reductions identified for amikacin and ticarcillin. *S. canis* demonstrated the most pronounced alterations, with significant MIC increases for seven antibiotics—neomycin, oxytetracycline, amikacin, gentamicin, chloramphenicol, tobramycin, and ofloxacin—and corresponding susceptibility reductions for several agents. *P. aeruginosa* exhibited minimal changes, with no statistically significant alterations in either MIC or susceptibility analysis. Ciprofloxacin consistently maintained stable MIC values and susceptibility classifications across all three species.

**Discussion:**

FML selectively modulated antimicrobial susceptibility in a species- and antibiotic-dependent manner, with a more restricted interaction profile than dexamethasone. Aminoglycosides demonstrated the greatest susceptibility variability, whereas ciprofloxacin maintained stable activity across all species, suggesting that it may represent a compatible option when concurrent FML use is clinically considered.

## 1 Introduction

Canine bacterial keratitis is a clinically significant condition in veterinary ophthalmology, and its management often requires concurrent antimicrobial and anti-inflammatory therapy (1, 2). Although topical corticosteroids are generally contraindicated in active bacterial keratitis, combination therapy is encountered in several clinical scenarios: in keratoconjunctivitis sicca (KCS) complicated by secondary bacterial infection, where long-term anti-inflammatory treatment is essential; during the resolution phase following bacterial keratitis, when residual corneal inflammation requires management; and in immune-mediated keratitis with concurrent bacterial involvement (3). In these settings, potent corticosteroids such as dexamethasone or prednisolone derivatives are frequently co-administered with antibiotics. However, their prolonged or repeated use is associated with well-documented adverse effects, including suppression of corneal wound healing, promotion of collagenase activity, immunosuppression in the presence of infection, and intraocular pressure (IOP) elevation—concerns that are particularly relevant when extended therapy is required (4).

In such clinical contexts, fluorometholone (FML) may be considered as an alternative corticosteroid offering a wider therapeutic safety margin. FML is a synthetic corticosteroid recognized for its limited penetration into the anterior chamber and a more favorable IOP profile compared with dexamethasone (5, 6). Experimental studies across veterinary species have confirmed this distinction: in a feline model of steroid-induced ocular hypertension, FML produced substantially smaller IOP elevations than dexamethasone or prednisolone acetate, and similar findings have been reported in equine studies (7, 8). These pharmacological properties make FML a relevant clinical option when the risks associated with higher-potency corticosteroids are a concern, and it has been used in selected veterinary patients for this reason.

Despite this clinical relevance, the potential for FML to alter antibiotic susceptibility has not been investigated in veterinary ocular pathogens. In human medicine, corticosteroid–antibiotic interactions have been shown to reduce the effectiveness of antibiotics including ciprofloxacin, gentamicin, and cefepime against clinically relevant bacterial isolates, and co-administration has been associated with treatment failure in some cases (9, 10). In veterinary ophthalmology, our previous study demonstrated that dexamethasone significantly altered the *in vitro* susceptibility of *Staphylococcus pseudintermedius*, *Streptococcus canis*, and *Pseudomonas aeruginosa*—three of the most prevalent pathogens in canine corneal infections—in an antibiotic class–dependent manner (11). However, because FML differs substantially from dexamethasone in its physicochemical properties, glucocorticoid receptor binding affinity, and degree of anterior chamber penetration, its interaction profile with antimicrobial agents cannot be extrapolated from prior dexamethasone-based findings and requires independent evaluation (4, 5).

Prior investigations have examined the interactions between topical corticosteroids and ophthalmic antibiotics in the context of corneal bacterial infections. A study evaluating four corticosteroids—including dexamethasone, FML, loteprednol, and prednisolone—against seven commonly used ophthalmic antibiotics demonstrated that corticosteroid co-exposure was associated with alterations in MIC values for selected antibiotic–bacterium combinations in pathogens relevant to corneal infection (12). These findings suggest that the antimicrobial efficacy of topical agents may be influenced by concurrent corticosteroid exposure, although the magnitude and specificity of such interactions appear to vary by drug and bacterial species. To date, however, systematic *in vitro* characterization of FML-specific interactions with a broad antibiotic panel in canine ocular pathogens remains limited.

Therefore, the present study aimed to evaluate the *in vitro* effects of FML on the antimicrobial susceptibility of the same three canine corneal pathogens, using the same clinical isolates and MIC testing platform as our previous dexamethasone investigation, though the steroid preparation method, concentration range, and statistical approach differed between the two studies (11). By testing 17 ophthalmic antibiotics across increasing FML concentrations, we sought to identify which antibiotic–FML combinations maintain stable antimicrobial activity and which are associated with clinically meaningful reductions in susceptibility, thereby providing experimental evidence to inform safer therapeutic decision-making in veterinary ophthalmology.

## 2 Materials and methods

### 2.1 Ocular isolates

From October 2022 to October 2023, samples were collected from dogs diagnosed with suspected bacterial keratitis at seven animal hospitals in various cities of Korea, using the same clinical isolate cohort and collection protocol as previously described in our dexamethasone–antibiotic interaction study (11). Bacterial growth was confirmed in 103 of these specimens. The sampled cases encompassed a wide range of clinical manifestations consistent with bacterial keratitis, including corneal ulceration, keratoconjunctivitis sicca complicated by secondary bacterial infection, keratomalacia, descemetocele formation, and corneal perforation. Among the cultured isolates, the three bacterial species most frequently recovered from corneal ulcers—*Staphylococcus pseudintermedius*, *Streptococcus canis*, and *Pseudomonas aeruginosa*—were selected for further analysis. Ten isolates from each species (n = 30 total) were included in the study and preserved at −80 °C using a microorganism preservation system (BactoBank Preservation System, Pulse Scientific Inc., Ontario, Canada) until testing (2022–2024).

### 2.2 Fluorometholone solutions

FML powder (Sigma-Aldrich, St. Louis, MO, catalog no. F9381-500MG) was used to prepare all experimental solutions. Target final concentrations (0.25, 0.5, and 1 mg/mL) were selected based on the labeled concentration of commercially available ophthalmic fluorometholone formulations, which are typically available at 0.1% (1 mg/mL) for clinical use. Representative products include FML® (Allergan, Irvine, CA), Flarex® (Alcon, Fort Worth, TX), and fluorometholone-containing combination formulations available in multiple countries.

A concentrated FML stock solution (75 mg/mL) was prepared by dissolving the weighed FML powder in dimethyl sulfoxide (DMSO; Merck, Darmstadt, Germany; CAS No. 67-68-5) under continuous mixing until complete solubilization was achieved. The stock solution was protected from light and stored under refrigerated conditions until use. To facilitate aqueous dilution and enhance solubility, a 15% (w/v) hydroxypropyl-*β*-cyclodextrin (HPβCD) solution [(2-Hydroxypropyl)-β-cyclodextrin; Sigma-Aldrich, catalog no. H107] was prepared in phosphate-buffered saline (PBS 1X, Phosphate Buffered Saline; Sigma-Aldrich) with gentle heating and continuous stirring until a clear solution was obtained.

Intermediate FML solutions were first prepared in the HPβCD carrier at four concentrations: 0 (control), 0.5, 1, and 2 mg/mL. These intermediate solutions were subsequently mixed with Mueller–Hinton broth (MHB, Thermo Scientific Inc., Waltham, MA, catalog no. T3462,) at a 1:1 ratio, yielding final working concentrations of 0, 0.25, 0.5, and 1 mg/mL for antimicrobial susceptibility testing. To maintain consistent solvent exposure across experimental groups, the volumes of stock solution and HPβCD were adjusted so that the final concentrations of DMSO and HPβCD remained comparable among all preparations. In the final test mixtures, the calculated DMSO concentrations ranged from approximately 0.33% to 1.35%, while HPβCD concentrations remained relatively constant at 7.3%–7.45% across all FML conditions. Previous antimicrobial susceptibility studies have demonstrated that DMSO concentrations below approximately 1%–2% do not significantly affect bacterial growth or MIC determinations, supporting its suitability as a solvent in *in vitro* assays (13). In addition, HPβCD is widely used as a pharmaceutical solubilizer with minimal inherent antimicrobial activity and low cytotoxicity (14).

### 2.3 Antimicrobial susceptibility testing

Thirty bacterial isolates were revived from −80 °C± 10 °C storage by thawing them at room temperature. They were then cultured on tryptic soy agar plates containing 5% sheep blood and incubated at 35 °C± 2 °C in a 5%–10% CO_2_ atmosphere for 24 to 48 h. Bacterial colonies were identified using the Matrix-Assisted Laser Desorption/Ionization Time-of-Flight (MALDI-TOF) method with the Microflex instrument. Each bacterial suspension was prepared in phosphate-buffered saline and adjusted to a 0.5 McFarland Standard using a nephelometer (DensiCHEK plus, BioMerieux, Marcy-l’Étoile, France) to ensure uniform colony density. Following this, 20 microliters of each suspension were transferred to standard Mueller-Hinton broth for testing against the target bacteria. To verify purity, a 1 mL sample of each suspension was plated on 5% sheep blood agar, streaked for isolation, and incubated at 35 °C± 2 °C for 16 to 24 h. Once confirmed as pure, susceptibility tests proceeded.

### 2.4 Preparation of broth cultures

For each bacterial isolate, a total of 11 mL of broth medium was allocated into two experimental sets. The first set, designated as the positive control, consisted of dispensing 0.25 mL of broth into four 2-mL microcentrifuge tubes, each preloaded with equal volumes of FML solution spanning concentrations from 0 to 2 mg/mL.

The second set, used for antimicrobial susceptibility testing, was prepared by distributing 1.5 mL of broth into ten 5-mL polypropylene tubes, each containing an equivalent volume of FML solution to generate the same concentration range (0–2 mg/mL). The control tubes were used to confirm bacterial viability, whereas the second set served as the experimental condition for susceptibility assessment. Because the broth and FML solutions were combined at a 1:1 ratio, the initial concentrations of both the bacterial inoculum and FML were adjusted accordingly and were effectively doubled prior to dilution in the assay plates.

### 2.5 Positive control plate

For each bacterial isolate, a blank plate with no antibiotics (Greiner 96 well plates, polypropylene, Greiner Bio-One.) was used. Each solution (broth-FML mixture) was manually pipetted, with 50 μL transferred into the eight wells of each column.

### 2.6 Antimicrobial susceptibility plates

Antimicrobial susceptibility testing was conducted using JOEYE2 microdilution plates optimized for ophthalmic applications (Thermo Scientific Inc., Waltham). For each bacterial isolate, intermediate broth–FML mixtures were first prepared at nominal concentrations of 0, 0.5, 1, and 2 mg/mL. Each intermediate mixture (50 μL) was manually dispensed into the 48 wells of the JOEYE2 plates, which were preloaded with serial dilutions of 17 ophthalmic antibiotics, including amikacin, cefazolin, ceftiofur, chloramphenicol, ciprofloxacin, doxycycline, erythromycin, gentamicin, moxifloxacin, neomycin, ofloxacin, oxytetracycline, polymyxin B, ticarcillin, tobramycin, and trimethoprim/sulfamethoxazole (T/S).

Following inoculation, the intermediate mixtures were diluted 1:1 with Mueller–Hinton broth within the assay system, resulting in final FML exposure concentrations of 0, 0.25, 0.5, and 1 mg/mL. Two plates were assigned to each isolate (*Staphylococcus pseudintermedius*, *Streptococcus canis*, and *Pseudomonas aeruginosa*; *n* = 10 per species). Plate 1 evaluated the intermediate concentrations of 0 and 0.5 mg/mL, corresponding to final concentrations of 0 and 0.25 mg/mL, respectively, whereas Plate 2 evaluated intermediate concentrations of 1 and 2 mg/mL, corresponding to final concentrations of 0.5 and 1 mg/mL.

Incubation conditions were determined according to Clinical and Laboratory Standards Institute (CLSI) recommendations. Plates inoculated with *Pseudomonas* species were incubated at 37 °C± 2 °C for 16–20 h, those containing *Streptococcus* species for 20–24 h, and those containing *Staphylococcus* species for approximately 24 h.

### 2.7 Data recording

Bacterial growth in antibiotic-free control wells was assessed visually using a manual plate reader (Scientific™ Sensititre™ Manual Viewer; Thermo Scientific Inc.). For the JOEYE2 susceptibility plates, the minimum inhibitory concentration (MIC) for each of the 17 tested antibiotics was defined as the lowest drug concentration that completely prevented visible bacterial growth. MIC values were subsequently categorized as susceptible, intermediate, resistant, or non-interpretable in accordance with established interpretive criteria outlined in CLSI VET01S, CLSI M100, and the European Committee on Antimicrobial Susceptibility Testing (EUCAST) guidelines (15, 16).

Isolates exhibiting intrinsic resistance, as defined by CLSI and EUCAST recommendations, were excluded from susceptibility analysis. Specifically, *Staphylococcus pseudintermedius* and *Streptococcus canis* were considered intrinsically resistant to polymyxin B, and *Pseudomonas aeruginosa* to chloramphenicol, oxytetracycline, ticarcillin, and trimethoprim/sulfamethoxazole (T/S), in accordance with Appendix B of the CLSI VET01S guideline (17, 18). In addition, antibiotic–isolate combinations for which established susceptibility breakpoints were not available were classified as non-interpretable and excluded from categorical analysis; bacitracin fell into this category across all three bacterial species. Gentamicin was excluded from categorical susceptibility analysis in *Streptococcus canis* as all isolates showed uniformly non-susceptible MIC values throughout the study, precluding Cochran’s *Q* testing; however, gentamicin MIC values were retained in the Friedman test analysis. The specific breakpoints and exclusion rationale for each antibiotic–species combination are summarized in Supplementary Table S1.

### 2.8 Statistical analysis

The Shapiro–Wilk test was used to assess the normality of the data. Because minimum inhibitory concentration (MIC) values were not normally distributed and represented ordinal dilution measurements, nonparametric statistical methods were applied. Because the same bacterial isolates (*n* = 10 per species) were evaluated across all fluorometholone concentrations (0, 0.25, 0.5, and 1 mg/mL), MIC data constituted repeated measures by design; therefore, the Friedman test was applied to assess overall differences in MIC across concentrations for each antibiotic–species combination (*n* = 17 antibiotics × 3 species). When overall significance was detected, pairwise comparisons between the baseline condition (0 mg/mL) and each fluorometholone concentration were performed using the Wilcoxon signed-rank test with Bonferroni correction for three comparisons. The false discovery rate was controlled using the Benjamini–Hochberg procedure applied across all antibiotics within each species. Median MIC values were calculated for each condition and reported in μg/mL.

Antimicrobial susceptibility outcomes were categorized as susceptible or non-susceptible (intermediate + resistant) according to established clinical breakpoints. Because the same isolates were tested across all concentrations, changes in susceptibility proportions were analyzed using Cochran’s *Q* test for overall comparisons, followed by McNemar’s test for pairwise comparisons between baseline and each FML concentration when appropriate.

All statistical analyses were performed using R version 4.4.0 (R Foundation for Statistical Computing, Vienna, Austria). A *p*-value < 0.05 was considered statistically significant.

## 3 Results

### 3.1 Positive control in antimicrobial susceptibility testing

All positive control wells demonstrated visible bacterial growth across the entire set of 30 isolates, with growth observed in all 32 wells per isolate (eight wells for each of the four FML concentration conditions). These findings indicate that fluorometholone alone, within the tested concentration range (0.5–2 mg/mL in the intermediate solutions), did not exert detectable antimicrobial activity.

### 3.2 Effect of fluorometholone on median MIC values in *Staphylococcus pseudintermedius*

**Figure 1:**
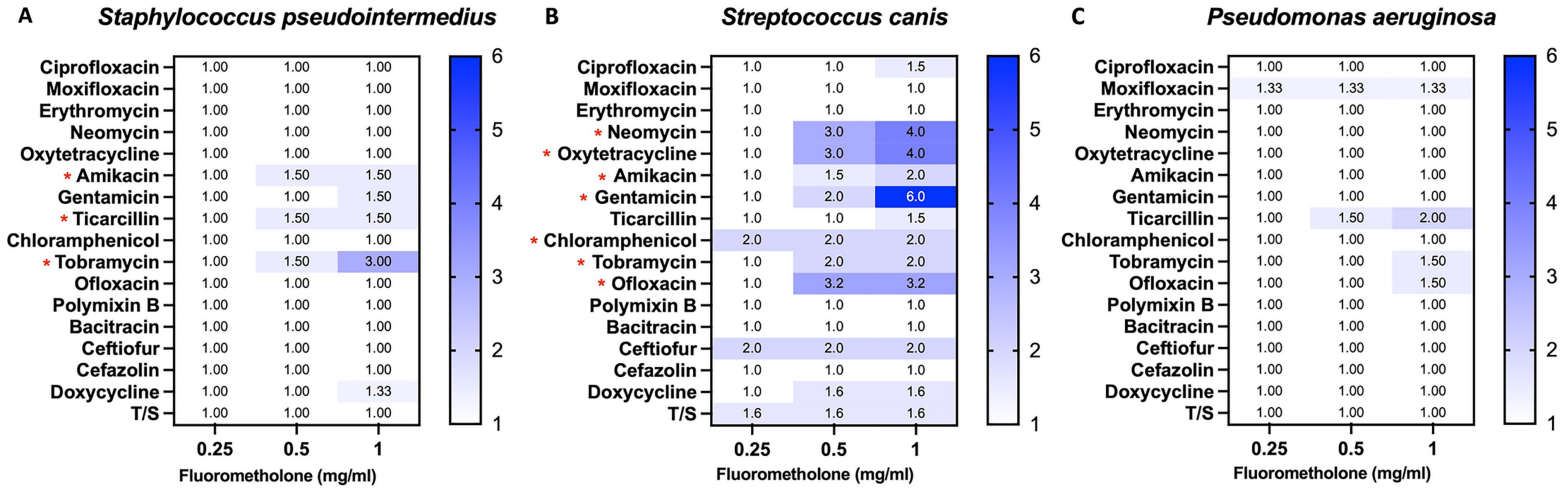
Heat maps illustrating fold changes in median minimum inhibitory concentrations (MICs) of ophthalmic antibiotics in the presence of fluorometholone (FML). Fold changes in median MIC values are displayed for each antibiotic relative to the control condition (0 mg/mL FML) across increasing FML concentrations (0.25, 0.5, and 1 mg/mL). Panels represent individual bacterial species: **(A)** *Staphylococcus pseudintermedius*, **(B)** *Streptococcus canis*, and **(C)** *Pseudomonas aeruginosa*. Color intensity corresponds to the magnitude of MIC fold change, with darker shading indicating greater increases relative to baseline. Antibiotic names marked with a red asterisk (*) indicate statistically significant overall differences across fluorometholone concentrations within each species (Friedman test with Benjamini–Hochberg correction, *p* < 0.05). T/S, trimethoprim/sulfamethoxazole.

In *Staphylococcus pseudintermedius* isolates, exposure to FML resulted in minimal changes in median MICs across most tested antibiotics (Figure 1A; Supplementary Figure S1). The majority of agents, including ciprofloxacin, moxifloxacin, erythromycin, neomycin, oxytetracycline, gentamicin, chloramphenicol, ofloxacin, polymyxin B, bacitracin, ceftiofur, cefazolin, doxycycline, and trimethoprim/sulfamethoxazole, showed stable MIC values across the evaluated fluorometholone concentrations (0.25–1 mg/mL).

Statistically significant increases in median MIC were identified for three antibiotics. Amikacin median MIC increased from 16 μg/mL at baseline to 24 μg/mL at 0.5 and 1 mg/mL (*p*_adj = 0.042). Ticarcillin showed a similar pattern, with median MIC rising from 16 μg/mL at baseline to 24 μg/mL at 0.5 and 1 mg/mL (*p*_adj = 0.042). Tobramycin exhibited the largest change, with median MIC increasing from 4 μg/mL at baseline to 6 μg/mL at 0.5 mg/mL and 12 μg/mL at 1 mg/mL (*p*_adj = 0.042). Post-hoc pairwise comparisons between baseline and individual concentrations did not reach significance after correction for any of these antibiotics, indicating that the overall concentration-dependent trend was detected but no single concentration could be identified as the threshold for significant MIC elevation.

### 3.3 Changes in antibiotic susceptibility for *Staphylococcus pseudintermedius*

FML exposure was associated with concentration-dependent changes in the proportion of susceptible *Staphylococcus pseudintermedius* isolates for several antibiotics (Table 1). Statistically significant reductions in susceptibility were identified for moxifloxacin, amikacin, and ticarcillin. Susceptibility to moxifloxacin decreased from 100% at 0 and 0.25 mg/mL to 80% at 0.5 mg/mL and 60% at 1 mg/mL (*p* = 0.024). Amikacin susceptibility declined from 100% at baseline to 80% at 0.25 mg/mL and further to 50% at both 0.5 and 1 mg/mL (*p* = 0.007). A comparable pattern was observed for ticarcillin, with susceptibility decreasing from 100% to 80% and subsequently to 50% at higher FML concentrations (*p* = 0.007).

**Table 1:** Percentage of antibiotic-susceptible *Staphylococcus pseudintermedius* isolates across increasing fluorometholone concentrations and statistical analysis results.

Fluorometholone	0 mg/ml	0.25 mg/mL	0.5 mg/mL	1 mg/mL	*p*-value
*Staphylococcus pseudointermedius*					
Ciprofloxacin	100%	100%	90%	90%	0.392
Moxifloxacin	100%	100%	80%	60%	0.024*
Erythromycin	30%	30%	30%	30%	N/A
Neomycin	30%	30%	20%	20%	0.392
Oxytetracycline	30%	20%	10%	10%	0.194
Amikacin	100%	80%	50%	50%	0.007*
Gentamicin	20%	20%	20%	20%	0.392
Ticarcillin	100%	80%	50%	50%	0.007*
Chloramphenicol	0%	0%	0%	0%	N/A
Tobramycin	70%	70%	50%	40%	0.061^†^
Ofloxacin	90%	90%	80%	80%	0.392
Polymixin B	0%	0%	0%	0%	N/A
Bacitracin	0%	0%	0%	0%	N/A
Ceftiofur	0%	0%	0%	0%	N/A
Cefazolin	100%	100%	80%	80%	0.112
Doxycycline	10%	10%	10%	20%	N/A
T/S	40%	40%	30%	30%	0.392

Susceptibility is expressed as the percentage of susceptible isolates (*n* = 10) of *Staphylococcus pseudintermedius* evaluated at fluorometholone concentrations of 0, 0.25, 0.5, and 1 mg/mL. Changes across concentrations were assessed using Cochran’s *Q* test. Antibiotics demonstrating statistically significant decreases in susceptibility are indicated in bold (*p < 0.05).

^†^Indicates a nonsignificant decreasing trend (0.05 ≤ *p* < 0.10). “N/A” denotes antibiotics showing no variation in susceptibility and therefore not included in the statistical analysis.

Tobramycin demonstrated a downward trend in susceptibility (70% at 0–0.25 mg/mL, 50% at 0.5 mg/mL, and 40% at 1 mg/mL), although this did not reach statistical significance (*p* = 0.061). In contrast, ciprofloxacin, neomycin, oxytetracycline, gentamicin, ofloxacin, cefazolin, doxycycline, and trimethoprim/sulfamethoxazole did not exhibit statistically meaningful changes in susceptibility across the tested FML concentrations. Several antibiotics, including erythromycin, chloramphenicol, polymyxin B, bacitracin, and ceftiofur, showed no variability in susceptibility and were therefore excluded from statistical analysis.

### 3.4 Effect of fluorometholone on median MIC values in *Streptococcus canis*

In *Streptococcus canis* isolates, exposure to FML was associated with statistically significant increases in median MICs for seven antibiotics: neomycin, oxytetracycline, amikacin, gentamicin, chloramphenicol, tobramycin, and ofloxacin (Figure 1B; Supplementary Figure S2). Neomycin demonstrated a marked elevation, with median MIC increasing from 4 μg/mL at baseline to 12 μg/mL at 0.5 mg/mL and 16 μg/mL at 1 mg/mL (*p*_adj = 0.018). Gentamicin showed a progressive increase from 2 μg/mL at baseline to 12 μg/mL at 1 mg/mL (*p*_adj = 0.005), with post-hoc comparison confirming a significant difference between baseline and 1 mg/mL. Tobramycin median MIC increased from 4 μg/mL at baseline to 8 μg/mL at both 0.5 and 1 mg/mL (*p*_adj = 0.005), with a significant difference also identified between baseline and 1 mg/mL. Oxytetracycline increased from 2 to 8 μg/mL, amikacin from 16 to 32 μg/mL, and ofloxacin from 0.6 to 1.92 μg/mL at the highest FML concentration, each reaching overall significance (*p*_adj = 0.043, 0.043, and 0.010, respectively), although pairwise comparisons with baseline did not reach significance after correction. Chloramphenicol showed an early increase from 16 μg/mL at baseline to 32 μg/mL at 0.25 mg/mL, which was maintained through 1 mg/mL (*p*_adj = 0.018), without significant post-hoc pairwise differences.

In contrast, ciprofloxacin, moxifloxacin, erythromycin, ticarcillin, polymyxin B, bacitracin, cefazolin, ceftiofur, doxycycline, and trimethoprim/sulfamethoxazole remained largely unchanged across the tested FML concentrations. Overall, FML exposure selectively influenced MIC values in *Streptococcus canis*, with the most pronounced effects observed for aminoglycosides and, to a lesser extent, other antibiotic classes.

### 3.5 Changes in antibiotic susceptibility for *Streptococcus canis*

The proportion of susceptible *Streptococcus canis* isolates demonstrated differential responses to increasing concentrations of FML (Table 2). Statistically significant reductions in susceptibility were identified for neomycin, oxytetracycline, and tobramycin. Neomycin susceptibility declined from 80% at baseline to 30% at 1 mg/mL (*p* = 0.029), while oxytetracycline decreased from 60% to 30% across the same concentration range (*p* = 0.046). Tobramycin showed the most pronounced reduction, with susceptibility decreasing from 60% at 0 mg/mL to 10% at 1 mg/mL (*p* = 0.010).

**Table 2:** Percentage of antibiotic-susceptible *Streptococcus canis* isolates across increasing fluorometholone concentrations and statistical analysis results.

Fluorometholone	0 mg/ml	0.25 mg/ml	0.5 mg/mL	1 mg/ml	*p*-value
*Streptococcus canis*					
Ciprofloxacin	60%	50%	50%	50%	0.572
Moxifloxacin	100%	90%	90%	80%	0.052^†^
Erythromycin	40%	40%	30%	30%	0.723
Neomycin	80%	60%	40%	30%	0.029*
Oxytetracycline	60%	60%	40%	30%	0.046*
Amikacin	60%	60%	40%	40%	0.572
Gentamicin	0%	0%	0%	0%	N/A
Ticarcillin	60%	60%	50%	40%	0.261
Chloramphenicol	0%	0%	0%	0%	N/A
Tobramycin	60%	50%	30%	10%	0.010*
Ofloxacin	60%	60%	40%	40%	0.572
Polymixin B	0%	0%	0%	0%	N/A
Bacitracin	0%	0%	0%	0%	N/A
Ceftiofur	50%	40%	30%	20%	0.392
Cefazolin	60%	60%	60%	60%	1.000
Doxycycline	30%	20%	10%	10%	0.392
T/S	60%	50%	50%	50%	0.801

Susceptibility is expressed as the percentage of susceptible isolates (*n* = 10) of *Streptococcus canis* evaluated at fluorometholone concentrations of 0, 0.25, 0.5, and 1 mg/mL. Changes across concentrations were assessed using Cochran’s *Q* test. Antibiotics demonstrating statistically significant decreases in susceptibility are indicated in bold (*p < 0.05).

^†^Indicates a nonsignificant decreasing trend (0.05 ≤ *p* < 0.10). “N/A” denotes antibiotics showing no variation in susceptibility and therefore not included in the statistical analysis.

Moxifloxacin exhibited a decreasing trend in susceptibility (100% at baseline to 80% at 1 mg/mL), although this did not reach statistical significance (*p* = 0.052). In contrast, ciprofloxacin, erythromycin, amikacin, ticarcillin, ofloxacin, ceftiofur, cefazolin, doxycycline, and trimethoprim/sulfamethoxazole did not show statistically meaningful changes in susceptibility across FML concentrations. Several antibiotics, including gentamicin, chloramphenicol, polymyxin B, and bacitracin, showed no measurable susceptibility throughout the study and were therefore excluded from statistical analysis.

### 3.6 Effect of fluorometholone on median MIC values in *Pseudomonas aeruginosa*

In *Pseudomonas aeruginosa* isolates, exposure to FML resulted in minimal alterations in median MICs across the tested antibiotics, with no statistically significant differences detected after correction (Figure 1C; Supplementary Figure S3). Most agents, including ciprofloxacin, erythromycin, neomycin, oxytetracycline, amikacin, gentamicin, chloramphenicol, polymyxin B, bacitracin, ceftiofur, cefazolin, doxycycline, and trimethoprim/sulfamethoxazole, exhibited stable MIC values across all tested FML concentrations.

Minor numerical increases were observed for a limited subset of antibiotics. Moxifloxacin median MIC increased from 0.75 μg/mL at baseline to 1.0 μg/mL at 0.25 mg/mL and remained stable thereafter. Ticarcillin showed a gradual increase from 16 μg/mL at baseline to 24 μg/mL at 0.5 mg/mL and 32 μg/mL at 1 mg/mL. Tobramycin and ofloxacin demonstrated modest increases at the highest FML concentration, with median MIC rising from 4 to 6 μg/mL and from 0.48 to 0.72 μg/mL, respectively. None of these variations reached statistical significance after correction, and no consistent dose-dependent pattern was identified. Overall, FML exposure did not produce statistically meaningful alterations in median MIC values in *Pseudomonas aeruginosa* isolates.

### 3.7 Changes in antibiotic susceptibility for *Pseudomonas aeruginosa*

Among the *Pseudomonas aeruginosa* isolates, changes in the proportion of susceptible isolates across increasing FML concentrations were limited and largely antibiotic-specific (Table 3). A statistically significant reduction in susceptibility was observed only for tobramycin, with the proportion of susceptible isolates decreasing from 90% at baseline to 60% at 0.5 mg/mL and further to 50% at 1 mg/mL (*p* = 0.0001). Notably, tobramycin did not show a statistically significant overall MIC increase in *Pseudomonas aeruginosa*; however, the significant reduction in susceptibility proportion suggests that categorical susceptibility shifts may occur even in the absence of a detectable median MIC change, likely reflecting isolate-level MIC shifts near the susceptibility breakpoint.

**Table 3:** Percentage of antibiotic-susceptible *Pseudomonas aeruginosa* isolates across increasing fluorometholone concentrations and statistical analysis results.

Fluorometholone	0 mg/ml	0.25 mg/ml	0.5 mg/ml	1 mg/ml	*p*-value
*Pseudomonas aeruginosa*					
Ciprofloxacin	90%	90%	90%	90%	N/A
Moxifloxacin	90%	70%	70%	70%	0.051^†^
Erythromycin	0%	0%	0%	0%	N/A
Neomycin	80%	80%	80%	80%	N/A
Oxytetracycline	0%	0%	0%	0%	N/A
Amikacin	90%	90%	90%	80%	0.262
Gentamicin	70%	70%	70%	70%	N/A
Ticarcillin	0%	0%	0%	0%	N/A
Chloramphenicol	0%	0%	0%	0%	N/A
Tobramycin	90%	90%	60%	50%	0.0001*
Ofloxacin	80%	80%	80%	80%	N/A
Polymixin B	40%	40%	30%	30%	0.262
Bacitracin	0%	0%	0%	0%	N/A
Ceftiofur	10%	10%	0%	0%	0.262
Cefazolin	10%	10%	10%	0%	0.262
Doxycycline	0%	0%	0%	0%	N/A
T/S	0%	0%	0%	0%	N/A

Susceptibility is expressed as the percentage of susceptible isolates (*n* = 10) of *Pseudomonas aeruginosa* evaluated at fluorometholone concentrations of 0, 0.25, 0.5, and 1 mg/mL. Changes across concentrations were assessed using Cochran’s *Q* test. Antibiotics demonstrating statistically significant decreases in susceptibility are indicated in bold (*p < 0.05).

^†^Indicates a nonsignificant decreasing trend (0.05 ≤ *p* < 0.10). “N/A” denotes antibiotics showing no variation in susceptibility and therefore not included in the statistical analysis.

Moxifloxacin demonstrated a decreasing trend in susceptibility, declining from 90% at 0 mg/mL to 70% at 0.25–1 mg/mL; however, this change did not reach statistical significance (*p* = 0.051). In contrast, ciprofloxacin maintained stable susceptibility (90%) across all FML concentrations and was not included in statistical analysis due to the absence of variability. Most other antibiotics, including amikacin, polymyxin B, ceftiofur, cefazolin, and ofloxacin, showed minor numerical fluctuations without statistically meaningful differences. Several agents (erythromycin, oxytetracycline, ticarcillin, chloramphenicol, bacitracin, doxycycline, and trimethoprim/sulfamethoxazole) exhibited uniformly low or absent susceptibility throughout the study and were therefore excluded from statistical testing.

## 4 Discussion

This study examined the *in vitro* effects of FML on antibiotic susceptibility and median MIC profiles in three major canine ocular pathogens. FML exposure produced antibiotic- and species-specific responses rather than uniform shifts in antimicrobial activity. In *Staphylococcus pseudintermedius*, significant increases in median MIC were identified for amikacin, ticarcillin, and tobramycin, while susceptibility proportions declined significantly for amikacin and ticarcillin. In *Streptococcus canis*, both median MICs and susceptibility were affected across a broader range of antibiotics, including aminoglycosides and several additional agents, whereas *Pseudomonas aeruginosa* exhibited minimal changes with no statistically significant alterations in either median MIC or susceptibility proportions. These findings indicate that FML-related modulation of antibiotic activity is selective and context-dependent, underscoring the need for cautious antibiotic selection in clinical scenarios where corticosteroid co-administration may be considered, such as immune-mediated keratitis with secondary bacterial involvement or the resolution phase of bacterial keratitis, rather than in active primary bacterial keratitis where corticosteroids remain contraindicated.

The bacterial species evaluated in this study—*Staphylococcus pseudintermedius*, *Streptococcus canis*, and *Pseudomonas aeruginosa*—represent the most frequently isolated pathogens in canine ulcerative and bacterial keratitis, collectively accounting for a substantial proportion of clinical cases reported in referral populations (1). These organisms readily colonize compromised corneal epithelium, activate host immune pathways, and contribute to progressive stromal inflammation and tissue degradation, thereby exacerbating corneal injury and delaying healing (19–21). Accordingly, clinical management of infectious keratitis typically relies on the concurrent administration of topical antimicrobial agents to eradicate pathogens and anti-inflammatory medications to modulate excessive inflammatory responses. This therapeutic overlap highlights the clinical relevance of investigating potential interactions between these drug classes. Although a diverse spectrum of microorganisms may colonize the ocular surface, focusing on these predominant pathogens provides a representative and clinically meaningful framework for evaluating drug–drug interactions under controlled experimental conditions.

Prior investigations have examined the interactions between topical corticosteroids and ophthalmic antibiotics in the context of corneal bacterial infections (12). A study evaluating four corticosteroids—including dexamethasone, FML, loteprednol, and prednisolone—against seven commonly used ophthalmic antibiotics demonstrated that corticosteroid co-exposure was associated with alterations in MIC values for selected antibiotic–bacterium combinations in pathogens relevant to corneal infection. These findings suggest that the antimicrobial efficacy of topical agents may be influenced by concurrent corticosteroid exposure, although the magnitude and specificity of such interactions appear to vary by drug and bacterial species. To date, however, systematic *in vitro* characterization of FML-specific interactions with a broad antibiotic panel in canine ocular pathogens remains limited.

In the present study, *Staphylococcus pseudintermedius* demonstrated selective alterations following FML exposure. Statistically significant increases in median MIC were identified for amikacin, ticarcillin, and tobramycin, suggesting that FML can elevate the antibiotic concentration required to inhibit bacterial growth for specific agents, even in the absence of a broadly suppressive effect. Susceptibility analysis further identified significant reductions for amikacin and ticarcillin, indicating that MIC elevations in these antibiotics were sufficient to produce categorical susceptibility shifts. In contrast, ciprofloxacin maintained stable MIC values and susceptibility profiles across all tested FML concentrations, whereas ofloxacin exhibited a significant MIC increase in *Streptococcus canis*, indicating that FML-related modulation in *Staphylococcus pseudintermedius* is selective rather than generalized.

*Streptococcus canis* demonstrated the most pronounced susceptibility alterations among the three evaluated species following FML exposure. Median MIC analysis revealed statistically significant increases for seven antibiotics: neomycin, oxytetracycline, amikacin, gentamicin, chloramphenicol, tobramycin, and ofloxacin. Among these, aminoglycoside-class agents showed the most substantial changes—gentamicin median MIC increased from 2 μg/mL at baseline to 12 μg/mL at 1 mg/mL, neomycin from 4 μg/mL to 16 μg/mL at 1 mg/mL, and tobramycin from 4 μg/mL to 8 μg/mL at 0.5 and 1 mg/mL. Ofloxacin also showed a notable increase from 0.6 μg/mL at baseline to 1.92 μg/mL at 0.5 and 1 mg/mL. The magnitude and breadth of these changes suggest that *Streptococcus canis* may be particularly susceptible to FML–antibiotic interactions across multiple antibiotic classes. In contrast, ciprofloxacin, moxifloxacin, erythromycin, ticarcillin, cefazolin, doxycycline, and trimethoprim/sulfamethoxazole maintained stable median MIC values across the tested FML concentrations.

In contrast to the Gram-positive isolates, *Pseudomonas aeruginosa* demonstrated minimal susceptibility alterations following FML exposure in the present study. Median MIC values remained largely stable across all tested antibiotics, and no statistically significant changes were identified in either median MIC analysis or categorical susceptibility testing. Minor numerical fluctuations were observed for a limited subset of agents, including moxifloxacin, ticarcillin, tobramycin, and ofloxacin, but these did not reach statistical significance after correction. These findings suggest that, under the tested conditions, FML exerts a limited modulatory effect on antimicrobial activity against *Pseudomonas aeruginosa*, and that this species may be comparatively less susceptible to FML-related antibiotic interactions than the Gram-positive pathogens evaluated in this study.

Lee et al. reported that FML broadly reduced the antibacterial activity of multiple antibiotics against human corneal pathogens, including *Staphylococcus aureus*, *Staphylococcus epidermidis*, *Streptococcus pneumoniae*, and *Pseudomonas aeruginosa*. Across these species, diminished antibacterial performance was observed for several commonly used agents, notably moxifloxacin, ofloxacin, neomycin, tobramycin, and polymyxin B. All five of these antibiotics were also evaluated in the present study. In contrast to the broadly suppressive effects reported in the human model, the present findings in canine ocular isolates demonstrated a more selective interaction profile. Partial concordance was observed for aminoglycosides—neomycin and tobramycin showed susceptibility reductions in both studies—and ofloxacin also exhibited significant MIC increases in *Streptococcus canis* in the present study. However, polymyxin B remained stable across all three canine species, and no statistically significant susceptibility alterations were identified for *Pseudomonas aeruginosa* under the tested conditions, contrasting with the suppressive pattern reported in the human model. Collectively, these observations highlight both shared and divergent FML–antibiotic interaction patterns between human and canine corneal pathogens and suggest that aminoglycosides may represent a particularly vulnerable antibiotic class, whereas the responses of fluoroquinolones and polymyxin B appear more species- and context-dependent (12).

In the present study, FML demonstrated a selective interaction profile rather than a uniform effect on antimicrobial susceptibility. Statistically meaningful changes were confined to a limited subset of antibiotic–species combinations, most notably involving aminoglycosides and selected agents such as ticarcillin and ofloxacin, whereas several antibiotics—including ciprofloxacin, moxifloxacin, and polymyxin B—maintained stable median MICs and susceptibility profiles across all three bacterial species. Susceptibility shifts were species-dependent, with the most pronounced and broadly distributed alterations observed in *Streptococcus canis*, antibiotic-specific MIC elevations in *Staphylococcus pseudintermedius*, and largely preserved stability in *Pseudomonas aeruginosa*. Together, these findings indicate that FML-related modulation of antimicrobial activity is context-dependent rather than broadly suppressive.

This selective interaction profile contrasts with our previous *in vitro* study evaluating dexamethasone, which used the same bacterial isolates and identical MIC testing platform. Although the two studies differed in steroid preparation method, tested concentration range, and statistical approach, the dexamethasone study demonstrated broader and more consistent reductions in antimicrobial susceptibility across multiple antibiotics and bacterial species. In the dexamethasone model, exposure was associated with increased median MICs and reduced susceptibility for several fluoroquinolones and aminoglycosides, including ciprofloxacin, ofloxacin, tobramycin, and neomycin, with these effects occurring across more than one bacterial species. In contrast, the present study demonstrates that FML exerts a more restricted influence on antimicrobial susceptibility. While aminoglycosides—including tobramycin, gentamicin, neomycin, and amikacin—showed the most consistent susceptibility alterations across species, fluoroquinolone responses were more variable: ciprofloxacin maintained stable median MICs and susceptibility classifications across all three species, whereas ofloxacin exhibited a significant MIC increase in *Streptococcus canis* but remained unaffected in *Staphylococcus pseudintermedius* and *Pseudomonas aeruginosa*. Additionally, polymyxin B and cefazolin, which showed susceptibility alterations in the dexamethasone study, remained largely unaffected by FML exposure across all three species in the present analysis. These findings suggest that corticosteroid–antibiotic interactions are not a uniform class effect but are strongly influenced by steroid-specific pharmacologic properties. Compared with dexamethasone, FML exhibits lower glucocorticoid receptor affinity, reduced anti-inflammatory potency, and more limited tissue penetration, which may attenuate its capacity to broadly modulate bacterial membrane function, intracellular antibiotic accumulation, or adaptive stress-response pathways (22).

The clinical relevance of *in vitro* susceptibility changes must be interpreted in the context of actual drug exposure on the ocular surface. FML ophthalmic formulations are administered as suspensions, resulting in limited and variable free-drug availability at the corneal surface. Following topical instillation, FML is subject to rapid dilution and clearance through tear turnover, blinking, and nasolacrimal drainage, leading to short contact times and fluctuating local drug concentrations (23). Consequently, the sustained exposure conditions modeled *in vitro* may not fully reflect the dynamic pharmacokinetic environment encountered during routine topical therapy. However, under certain clinical circumstances, cumulative or prolonged steroid exposure may occur, including frequent dosing schedules, long-term anti-inflammatory treatment, postoperative management, or compromised tear clearance in inflamed eyes. In such settings, repeated dosing could transiently elevate local steroid concentrations and extend exposure duration, potentially increasing the likelihood of steroid–antibiotic interactions beyond what would be expected during standard dosing regimens. Therefore, while the selective and species-dependent susceptibility shifts observed in this study suggest a limited interaction potential under typical clinical use, caution may be warranted in scenarios involving intensified or prolonged FML administration, particularly when aminoglycoside-based regimens are employed.

Overall, the present study demonstrates that FML can interact with certain antibiotics and alter antimicrobial susceptibility in a drug- and species-dependent manner, underscoring the need for careful antibiotic selection when topical corticosteroids are used in the treatment of canine bacterial keratitis. Importantly, across all three bacterial species examined, ciprofloxacin consistently exhibited stable median MIC values and preserved susceptibility profiles, suggesting a relatively low likelihood of interaction with FML under the experimental conditions of this study. In the limited clinical scenarios where concurrent use of an antibiotic and a lower-potency corticosteroid such as FML may be considered—for example, in immune-mediated ocular surface disease with concurrent bacterial colonization—ciprofloxacin may represent a more pharmacologically compatible option based on the present *in vitro* findings alone. It should be emphasized, however, that corticosteroid use in active bacterial keratitis remains contraindicated, and the present findings are intended to inform antibiotic selection only in the specific contexts where such combinations are clinically justified.

In contrast, greater variability and statistically meaningful susceptibility changes were observed for several antibiotics, particularly among aminoglycosides—including amikacin, gentamicin, neomycin, and tobramycin—as well as ticarcillin and ofloxacin in specific bacterial species. These agents may therefore warrant more cautious use or closer consideration when combined with topical FML. Although the present findings are derived from an *in vitro* model and their direct clinical applicability remains uncertain, they highlight the importance of incorporating potential steroid–antibiotic interactions into antimicrobial selection strategies for canine bacterial keratitis. The present study included ten isolates per bacterial species, which, while consistent with our previous dexamethasone investigation, limits the statistical power of individual comparisons and may not fully capture the diversity of clinical isolates encountered in practice. These findings should therefore be interpreted as exploratory and require validation in larger isolate cohorts. Further *in vivo* studies are needed to clarify the clinical relevance of these interactions and to refine evidence-based treatment guidelines.

## Data Availability

The original contributions presented in the study are included in the article/Supplementary material, further inquiries can be directed to the corresponding author.

## Supplementary material

SUPPLEMENTARY FIGURE S1 Effects of fluorometholone on median minimum inhibitory concentrations of ophthalmic antibiotics in *Staphylococcus pseudintermedius*. Each dot represents an individual isolate (*n* = 10). Horizontal bars indicate median values. Fluorometholone concentrations of 0, 0.25, 0.5, and 1 mg/mL are represented by dark blue, light green, medium green, and dark green dots, respectively. Antibiotics with statistically significant overall differences across fluorometholone concentrations are indicated by an asterisk (*) in the title and highlighted with a green border (Friedman test with Benjamini–Hochberg correction, *p* < 0.05). T/S, trimethoprim/sulfamethoxazole.

SUPPLEMENTARY FIGURE S2 Effects of fluorometholone on median minimum inhibitory concentrations of ophthalmic antibiotics in *Streptococcus canis*. Each dot represents an individual isolate (*n* = 10). Horizontal bars indicate median values. Fluorometholone concentrations of 0, 0.25, 0.5, and 1 mg/mL are represented by dark blue, light green, medium green, and dark green dots, respectively. Antibiotics with statistically significant overall differences across fluorometholone concentrations are indicated by an asterisk (*) in the title and highlighted with a green border (Friedman test with Benjamini–Hochberg correction, *p* < 0.05). The dagger symbol (†) indicates a specific fluorometholone concentration at which a significant difference from baseline was identified by post-hoc Wilcoxon signed-rank test with Bonferroni correction (*p* < 0.05). T/S, trimethoprim/sulfamethoxazole.

SUPPLEMENTARY FIGURE S3 Effects of fluorometholone on median minimum inhibitory concentrations of ophthalmic antibiotics in *Pseudomonas aeruginosa*. Each dot represents an individual isolate (*n* = 10). Horizontal bars indicate median values. Fluorometholone concentrations of 0, 0.25, 0.5, and 1 mg/mL are represented by dark blue, light green, medium green, and dark green dots, respectively. No statistically significant differences were identified for any antibiotic across the tested fluorometholone concentrations (Friedman test with Benjamini–Hochberg correction). T/S, trimethoprim/sulfamethoxazole.
