# Supplementary figures and images for: Fluorometholone–antibiotic interactions in canine ocular bacteria: in vitro susceptibility changes in common corneal infection pathogens
Source: Front Vet Sci. 2026 Jun 10;13:1860758. doi: 10.3389/fvets.2026.1860758 (PMC13290699; doi:10.3389/fvets.2026.1860758)

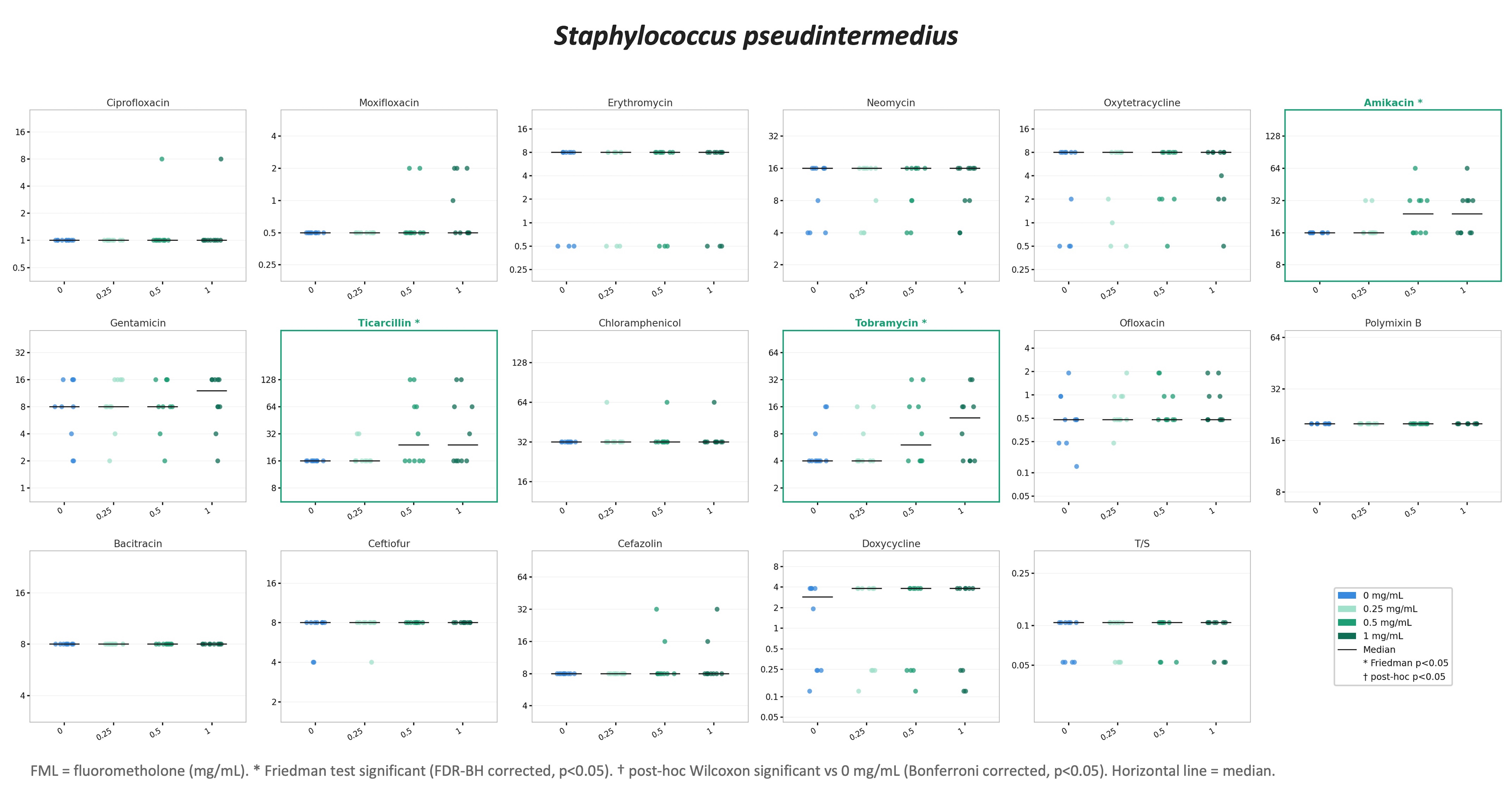

Supplement: SUPPLEMENTARY FIGURE S1 — Effects of fluorometholone on median minimum inhibitory concentrations of ophthalmic antibiotics in Staphylococcus pseudintermedius. Each dot represents an individual isolate (n = 10). Horizontal bars indicate median values. Fluorometholone concentrations of 0, 0.25, 0.5, and 1 mg/mL are represented by dark blue, light green, medium green, and dark green dots, respectively. Antibiotics with statistically significant overall differences across fluorometholone concentrations are indicated by an asterisk (*) in the title and highlighted with a green border (Friedman test with Benjamini–Hochberg correction, p < 0.05). T/S, trimethoprim/sulfamethoxazole. [file Image_1.jpeg]

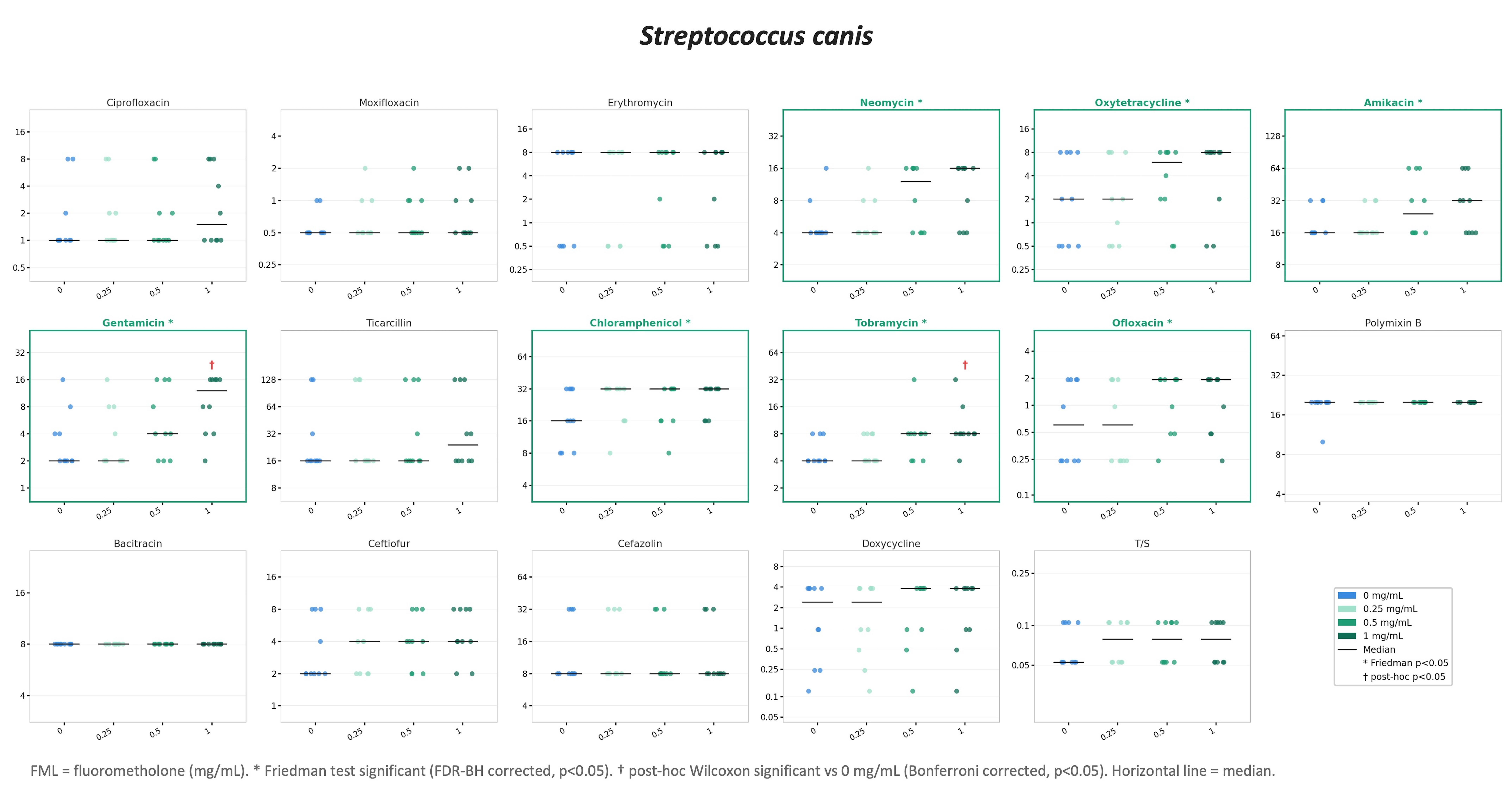

Supplement: SUPPLEMENTARY FIGURE S2 — Effects of fluorometholone on median minimum inhibitory concentrations of ophthalmic antibiotics in Streptococcus canis. Each dot represents an individual isolate (n = 10). Horizontal bars indicate median values. Fluorometholone concentrations of 0, 0.25, 0.5, and 1 mg/mL are represented by dark blue, light green, medium green, and dark green dots, respectively. Antibiotics with statistically significant overall differences across fluorometholone concentrations are indicated by an asterisk (*) in the title and highlighted with a green border (Friedman test with Benjamini–Hochberg correction, p < 0.05). The dagger symbol (†) indicates a specific fluorometholone concentration at which a significant difference from baseline was identified by post-hoc Wilcoxon signed-rank test with Bonferroni correction (p < 0.05). T/S, trimethoprim/sulfamethoxazole. [file Image_2.jpeg]

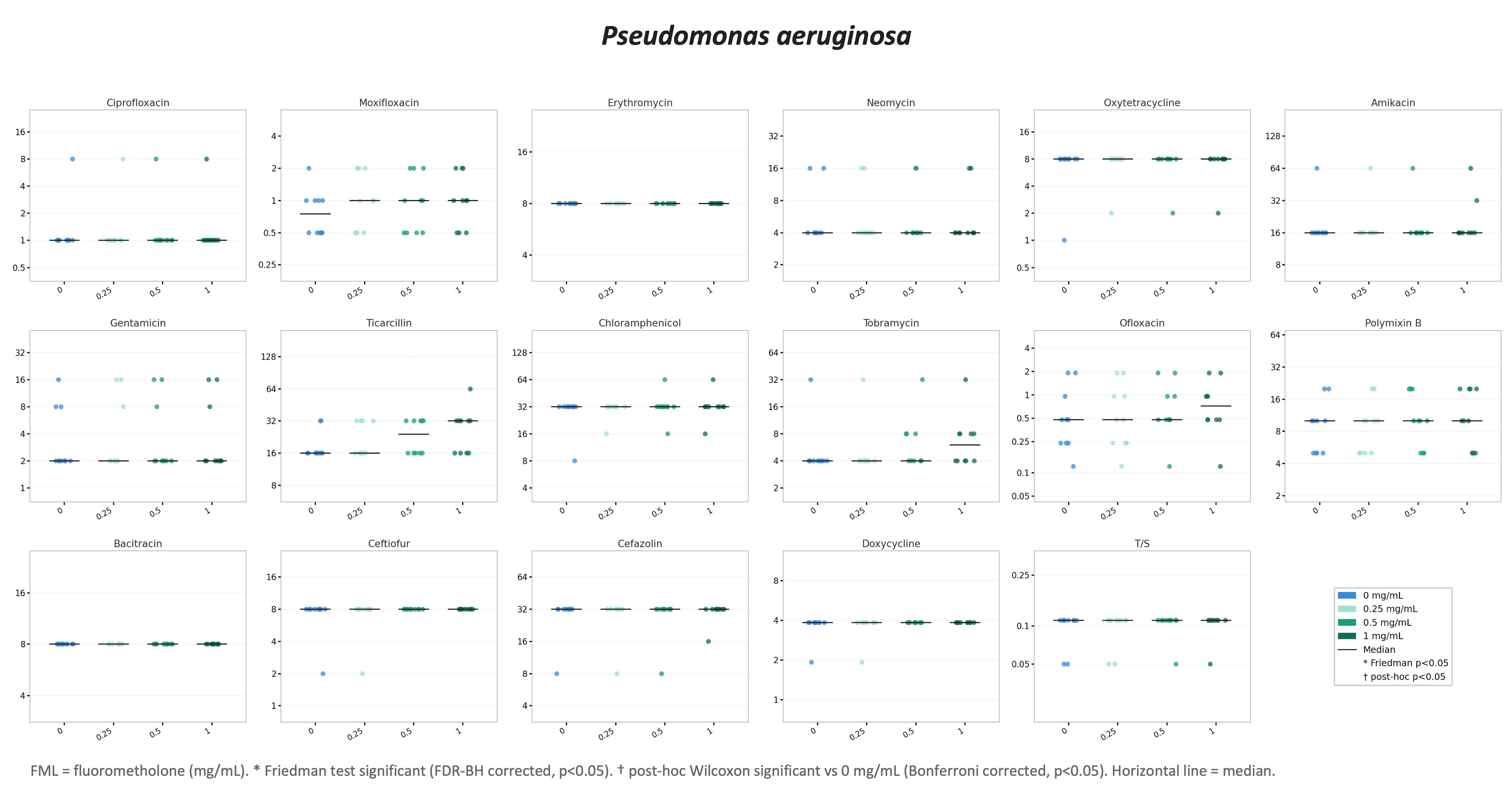

Supplement: SUPPLEMENTARY FIGURE S3 — Effects of fluorometholone on median minimum inhibitory concentrations of ophthalmic antibiotics in Pseudomonas aeruginosa. Each dot represents an individual isolate (n = 10). Horizontal bars indicate median values. Fluorometholone concentrations of 0, 0.25, 0.5, and 1 mg/mL are represented by dark blue, light green, medium green, and dark green dots, respectively. No statistically significant differences were identified for any antibiotic across the tested fluorometholone concentrations (Friedman test with Benjamini–Hochberg correction). T/S, trimethoprim/sulfamethoxazole. [file Image_3.jpeg]
