# Supplementary material for: Fluorometholone–antibiotic interactions in canine ocular bacteria: in vitro susceptibility changes in common corneal infection pathogens
Source: Front Vet Sci. 2026 Jun 10;13:1860758. doi: 10.3389/fvets.2026.1860758 (PMC13290699; doi:10.3389/fvets.2026.1860758)
Supplement: Supplementary file 4 [file Table_1.docx]

**Supplementary Table 1.** Antimicrobial susceptibility breakpoints (µg/mL) used for interpretation in this study, according to CLSI VET01S, CLSI M100, and EUCAST guidelines.

| **Antibiotic** | **Staphylococcus pseudintermedius** | | | **Streptococcus canis** | | | **Pseudomonas aeruginosa** | | |
| --- | --- | --- | --- | --- | --- | --- | --- | --- | --- |
|  | **S** | **I** | **R** | **S** | **I** | **R** | **S** | **I** | **R** |
| Amikacin | 4 | 8 | 16 | 4 | 8 | 16 | 4 | 8 | 16 |
| Bacitracin | Non-interpretable | | | Non-interpretable | | | Non-interpretable | | |
| Cefazolin | 2 | 4 | 8 | 2 | 4 | 8 | 8 | 16 | 32 |
| Ceftiofur | 2 | 4 | 8 | 2 | 4 | 8 | 8 | 16 | 32 |
| Chloramphenicol | 8 | 16 | 32 | 4 | 8 | 16 | Intrinsic resistance | | |
| Ciprofloxacin | 1 | 2 | 4 | 0.5 |  | 4 | 0.001 |  | 0.5 |
| Doxycycline | 0.12 | 0.25 | 0.5 | 0.25 | 0.5 | 1 | 0.12 | 0.25 | 0.5 |
| Erythromycin | 0.5 |  | 8 | 0.25 | 0.5 | 1 | 8 | 16 | 32 |
| Gentamicin | 4 | 8 | 16 | Intrinsic resistance | | | 2 | 4 | 8 |
| Moxifloxacin | 0.5 | 1 | 2 | 1 | 2 | 4 | 1 | 2 | 4 |
| Neomycin | 4 | 8 | 16 | 4 | 8 | 16 | 4 | 8 | 16 |
| Ofloxacin | 1 | 2 | 4 | 2 | 4 | 8 | 2 | 4 | 8 |
| Oxytetracycline | 0.25 | 0.5 | 1 | 2 | 4 | 8 | Intrinsic resistance | | |
| Polymyxin B | Intrinsic resistance | | | Intrinsic resistance | | |  | 2 | 4 |
| Ticarcillin | 0.25 |  | 0.5 | 0.25 |  |  | Intrinsic resistance | | |
| Tobramycin | 4 | 8 | 16 | 4 | 8 | 16 | 4 | 8 | 16 |
| T/S | 2/38 |  | 4/76 | 0.5/9.5 | 1/19–2/38 | 4/76 | Intrinsic resistance | | |

S, susceptible; I, intermediate; R, resistant; T/S, trimethoprim/sulfamethoxazole. Non-interpretable indicates antibiotic–species combinations for which established breakpoints were not available; these were excluded from categorical analysis. Intrinsic resistance indicates combinations excluded per CLSI and EUCAST recommendations.
